# Knowledge and Attitude Towards Botulinum Toxin Use in Cosmetic Injections Among the Arar Population in Saudi Arabia: A Cross-Sectional Study

**DOI:** 10.7759/cureus.70694

**Published:** 2024-10-02

**Authors:** Bandar T Alenezi, Rahma Hamayun, Anshoo Agarwal, Dalia Aqeel J Alanazi, Amzan Mohammad A Alruwaili, Wateen Ali A Alenezi, Ahad Khalifah M Alanazi

**Affiliations:** 1 Department of Pharmacology, Faculty of Medicine, Northern Border University, Arar, SAU; 2 Center for Health Research, Northern Border University, Arar, SAU; 3 Department of Pathology, Faculty of Medicine, Northern Border University, Arar, SAU; 4 Department of Medicine, Faculty of Medicine, Northern Border University, Arar, SAU

**Keywords:** adverse effects, botox injections, botulinum neurotoxin, health education & awareness, kingdom of saudi arabia (ksa)

## Abstract

Background

The use of botulinum toxin (BT), commonly known as Botox, has become increasingly popular for cosmetic purposes, particularly in treating wrinkles and facial rejuvenation. While the efficacy of botulinum toxin in achieving desired aesthetic outcomes is well-established, it is also important to consider the level of public knowledge and awareness regarding this potent neurotoxin. This study investigates the knowledge and attitude towards botulinum toxin use in cosmetic injections in the Arar population.

Methods

This cross-sectional study utilized a self-report questionnaire distributed via social media platforms, employing randomized sampling from May to July 2024. The target population included adults aged 18 and older, regardless of gender, living in the city of Arar, Saudi Arabia. The data were collected through an online self-administered survey using a Google Forms questionnaire template, which was translated into the appropriate language. The collected data was analyzed using descriptive and inferential statistics.

Results

A total of four hundred and ten (410) participants responded to this study. The maximum number of participants, 149 (36.3%), were in the age group of 20-25 years, while the minimum number of participants, 6 (1.5%), was in the age group of 50-55 years. The results also showed that 341 (83.2%) of participants were females and 69 (16.8%) were males; 262 (63.9%) participants were aware of Botox treatment used in cosmetic clinics. Concerning the most prevalent non-surgical cosmetic treatments, 29 (7%) answered dental procedures, and 19 (4.6%) mentioned its use in pain.

Conclusion

This study found that 262 (63.9%) of the study population showed knowledge about botulinum toxins used in cosmetic injections. The majority of them were young individuals, females, or unmarried people. Only 83 (20%) were aware of the adverse effects of botulinum toxin. This low level of awareness about the potential risks associated with the use of botulinum toxins for cosmetic purposes is concerning and suggests the need for improved education and information dissemination.

## Introduction

Known by many names, including "miracle poison," botulinum toxin (BT) is among the deadliest biological compounds on the planet [1]. It is a neurotoxin produced by the Gram-positive, anaerobic, spore-forming bacterium Clostridium botulinum, commonly found in plants, soil, water, and the digestive systems of animals. Kassir et al. first demonstrated the effectiveness of botulinum toxin type A in treating strabismus in humans [2]. Later on, botulinum toxin was licensed to treat a wide range of illnesses, including several spasticity disorders [1]. At the moment, practically all medical subspecialties employ it. Botox® (botulinum toxin-A) was authorized by the FDA in 2002 for the temporary treatment of glabellar forehead frown lines. To date, at least seven different serotypes of neurotoxins have been identified. These include botulinum toxins A, B, C, D, E, F, and G; however, only the first two are currently utilized in medicine. Many are drawn to it even after learning about its negative consequences to preserve their beauty [3,4]. The physiologically active part of BT, botulinum neurotoxin (BNT), is a big double-stranded protein that may be affected by a variety of physical and chemical factors. Since all BT type A (BT-A) medications are kept in powder form, their shelf life ranges from 24 to 36 months. The recommended shelf life is shortened to 8-24 hours after reconstitution [5]. The phrase "botulinum toxin," which is short for "Botox," is where the name originated [6].

To be able to use a toxin as a drug, it must be isolated, purified, and then stabilized. All serotypes of botulinum toxin interfere with the process of neural transmission. They do this by blocking the release of acetylcholine (ACh), which is the primary neurotransmitter responsible for conveying signals from nerves to muscles at the neuromuscular junction. This inhibition prevents muscle contraction, leading to paralysis. When botulinum toxin is injected into muscle tissue (a method known as intramuscular administration), it specifically targets the neuromuscular junction. Here, it acts on the presynaptic motor neurons, inhibiting them from releasing acetylcholine [7]. As a result, the muscles do not receive the signals needed to contract, leading to a temporary paralysis of those muscle fibers. Botulinum toxins have effects at four main locations within the body. The neuromuscular junction is the primary site where nerve cells communicate with muscles, crucial for voluntary movements. It also acts on autonomic ganglia, postganglionic parasympathetic nerve endings, and postganglionic sympathetic nerve endings. The ability of botulinum toxin to induce paralysis has made it useful in various medical and cosmetic applications. For instance, it is commonly used to treat conditions like strabismus (crossed eyes) and muscle spasms and, more widely known, for cosmetic procedures to reduce wrinkles by temporarily paralyzing the underlying muscles [8]. 

There are two preparations of botulinum toxin A: Disport® and Botox®. Botulinum toxin type A for medicinal purposes is sold as botulinum toxin A. Botox A is used to treat various medical conditions, primarily in dermatology, and for cosmetic purposes. The first Botox product on the market was based on botulinum toxin A [9]. A noteworthy aspect of BoNT/A1 is its little effect, even at short distances from the site of injection, which guarantees excellent outcomes in most clinical applications [10]. However, there is a chance of local diffusion or leakage into the systemic circulation, which is determined by a variety of parameters such as injection volume and speed, dosage, and injection location, which could cause ptosis, a heavy brow, or a frozen face in facial aesthetics [11-15].

It is occasionally employed as a substitute for surgical procedures [16]. Injecting botulinum toxin into the major muscles of both the upper and lower limbs in children with cerebral palsy leads to a consistent reduction in muscle activity [17] and is useful in treating achalasia [18]. Botulinum toxin works effectively for several autonomic illnesses that cause gland hypersecretion, such as ptyalism or gustatory sweating, which frequently arise following parotid gland surgery [19,20]. Botulinum toxin remains the most popular cosmetic surgery among both men and women.

However, to date, knowledge, attitudes, and practices about the use of botulinum toxin in cosmetic injections in the Arar population remain unexplored.

## Materials and methods

Study design and data collection

A population-based cross-sectional study was conducted in Arar, Saudi Arabia, from May to July 2024. A pre-validated and self-structured questionnaire was distributed via social media platforms with randomized sampling. The study's target population comprises adults aged 18 and above from the Arar city, including both genders of varying age groups, marital status, and educational backgrounds.

The selection of items for the questionnaire was informed by a thorough literature review (Appendices, Tables 5-7) [21]. The questionnaire had three sections. The first section contained personal data (age, gender, marital status, residency, family income, parents, education, and job). Two further sections focused on information related to knowledge and attitudes towards the use of botulinum toxin in cosmetic injections in this study group. The data were collected through an online self-administered questionnaire in both Arabic and English languages to target the diversity of the study population.

Sample size determination

The minimum required sample size for this study was calculated using Open Epi Version 3.0 (Dean AG, Sullivan KM, Soe MM. OpenEpi: Open Source Epidemiologic Statistics for Public Health, Version. www.OpenEpi.com, updated 2013/04/06), considering several factors: an estimated population size (as reported by the General Authority for Statistics), a confidence interval (CI) of 95%, and an expected frequency of 50%. The resulting sample size was determined to be 385 participants. To accommodate potential data loss, the total sample size was adjusted to 400 participants. This study was approved by the Bioethics Committee of Northern Border University HAP-09-A-043, under approval number (48/24/H).

Statistical analysis

The data were collected, reviewed, and entered into IBM SPSS Statistics for Windows, Version 21.0 (released 2012; IBM Corp., Armonk, New York, United States). The collected data were analyzed using descriptive and inferential statistics. All statistical methods were two-tailed, with a significance level (alpha) set at 0.05, considering results significant if the p-value was less than or equal to 0.05. Descriptive analysis included frequency distributions and percentages for various study variables, such as participants' personal information and cosmetic procedures experienced by them and their families. Cross-tabulation was employed to show factors related to participants' knowledge of cosmetic procedures, with statistical significance assessed using the Pearson chi-squared test. For instances with small frequency distributions, an exact probability test was utilized.

## Results

A total of four hundred and ten (410) participants responded to this study. The demographic characteristics of the study participants have been presented in Table 1. The maximum number of participants, 149 (36.3%), were in the age group of 20-25 years, while the minimum number of participants, 6 (1.5%), was in the age group of 50-55 years. The results also showed that 341 (83.2%) of participants were females and 69 (16.8%) were males. The maximum number of participants, 167 (40.7%), belong to the 51-60 kg weight group, and the lowest number of participants, 3 (0.7%), belong to the less than 40 kg weight group. In a similar pattern, a maximum number of participants, 231 (56.3%), had a height of 161-170 cm. The maximum number of participants, 385 (93.6%), were Saudi nationals, while a small portion of participants, 25 (6.1%), were non-Saudi nationals. Most participants, 271 (66.1%), were bachelors, whereas 262 (63.9%) were students. Similarly, the results showed that 276 (67.3%) of participants were single (Table 1).

**Table 1:** Sociodemographic data of study participants Data are presented as frequency (N) and percentages (%)

Characteristics	Frequency (N)	Percent (%)
Age groups (years)		
15–20	119	29.0
20–25	149	36.3
25–30	29	7.1
30–35	33	8.0
35–40	22	5.4
40–45	27	6.6
45–50	25	6.1
50–55	6	1.5
Gender		
Female	341	83.2
Male	69	16.8
Weight (kg)		
40–45	45	11.0
51–60	167	40.7
61–70	96	23.4
71–80	50	12.2
Less than 40	3	0.7
More than 80	49	12.0
Height (cm)		
141–150	32	7.8
151–160	231	56.3
161–170	117	28.5
More than 170	30	7.3
Nationality		
Non-Saudi	25	6.1
Saudi	385	93.9
Education level		
Bachelor	271	66.1
Doctorate	21	5.1
Higher Secondary	42	10.2
Master	18	4.4
Secondary	58	14.1
Occupation		
Employee	107	26.1
Faculty	3	0.7
Housewife	5	1.2
Private	29	7.1
Self-employed	4	1.0
Student	262	63.9
Marital status		
Divorced	4	1.0
Married	118	28.8
Single	276	67.3
Widower/widow	12	2.9

Thirty questions were asked to know the knowledge and attitude of participants towards Botox toxins. The results showed that 84.1% of participants (345) did not plan to have or undergo Botox treatment. In response to a question, do you know that botulinum toxin is one of the most poisonous biological substances known? 154 (37.6%) of the participants replied that they didn’t know, and 143 (34.9%) answered no. In contrast, 63.9% (262) of the participants were aware of the use of Botox treatment in cosmetic clinics. Concerning the most prevalent non-surgical cosmetic treatments, 29 (7%) answered dental procedures, and 19 (4.6%) mentioned their use in pain (Table 2).

**Table 2:** Knowledge and attitude towards Botox toxins usage among Arar population in Saudi Arabia Data are presented as frequency (N) and percentages (%)

Characteristics	Frequency (N)	Percent (%)
Do you plan to have or have undergone Botox treatment?		
No	345	84.1
Yes	65	15.9
Do you know that botulinum toxin is one of the most poisonous biological substances known?		
I don't know	154	37.6
No	143	34.9
Yes	113	27.6
Do you know about Botox treatment is used in cosmetic clinics?		
I don't know	32	7.8
No	116	28.3
Yes	262	63.9
Have you or any of your family member has taken Botox treatment for any type of disease?		
I don't know	17	4.1
No	336	82.0
Yes	57	13.9
If, yes please mention the medical condition for which Botox treatment was used:		
Total responses	324	79.0
Autonomic disorders	4	1.0
Autonomic disorders/dental treatment	2	0.5
Autonomic disorders/urologic pathologic conditions	2	0.5
Dental treatment	29	7.1
Dystonia	5	1.2
Dystonia/dental treatment	3	0.7
Dystonia/spasticity/autonomic disorders/urologic pathologic conditions/pain/gastroenterology disorders	5	1.2
Dystonia/spasticity/pain	5	1.2
Pain	19	4.6
Pain/dental treatment	1	0.2
Spasticity	6	1.5
Spasticity/gastroenterology disorders	2	0.5
Spasticity/pain	3	0.7
Have you or any of your family member has used Botox treatment to reduce all wrinkles in all skin areas of the face and the neck?		
I don't know	18	4.4
No	263	64.1
Yes	129	31.5
Do you know that the best results are obtained when Botox is combined with filler substances to correct non dynamic aspects of the skin lines?		
I don't know	115	28.0
No	216	52.7
Yes	79	19.3

Regarding the awareness of the adverse effects of botulinum toxin and misuse, it was found that 76.3% (313) of the participants were well aware of the use of botulinum toxin injections in the presence of a dermatologist. Two hundred and forty-nine (60.7%) agreed to the high cost of these injections. When asked about the side effects of botulinum toxin injections, only 33.7% (138) were aware of it. Regarding the prohibition of the use of botulinum toxin injections during pregnancy, only 53.2% (218) of the population knew it should not be used in pregnancy. Similarly, 50% (205) were aware that they should avoid these injections during lactation. 36.3% (149) of the population knew that weakness induced by injection with botulinum toxin usually lasts about three months (Table 3).

**Table 3:** Awareness of Botulinum toxins usage among the Arar population in Saudi Arabia Data are presented as frequency (N) and percentages (%)

Characteristics	Frequency (N)	Percent (%)
Are you aware of how to safely dispose of leftover needles after using them?		
I don't know	74	18.0
No	180	43.9
Yes	156	38.0
Are you aware that botulinum toxins and vials need to be stored according to the protocol approved by their manufacturer?		
I don't know	65	15.9
No	191	46.6
Yes	154	37.6
Do you think that buying needles without a prescription is against the law?		
I don't know	30	7.3
No	45	11.0
Yes	335	81.7
Do you think that those who wish to take Botox injections should contact a dermatologist instead of non-specialized workers in cosmetic centers?		
I don't know	32	7.8
No	65	15.9
Yes	313	76.3
Are you aware of the high cost of Botox treatment?		
I don't know	46	11.2
No	115	28.0
Yes	249	60.7
Are you aware that the medicine starts showing the desired results after two to three weeks of Botox treatment?		
I don't know	79	19.3
No	120	29.3
Yes	211	51.5
Are you aware that patients who have frequent booster injections seem to have a higher risk of developing antibodies and tolerance?		
I don't know	124	30.2
No	159	38.8
Yes	127	31.0
Are you aware that Bruising can occur, particularly if a small vein is lacerated or a patient is taking aspirin, vitamin E, or NSAIDs?		
I don't know	100	24.4
No	161	39.3
Yes	149	36.3
Do you know these Botox injections can be repeated to maintain the desired results?		
I don't know	50	12.2
No	63	15.4
Yes	297	72.4
Do you know that there is a possibility of local diffusion or potential leakage of Botulinum toxin into the systemic circulation?		
I don't know	107	26.1
No	153	37.3
Yes	150	36.6
Do you know that local Botulinum toxin injections could cause ptosis (droopy eyelids), a heavy brow, or a frozen face in facial aesthetics?		
I don't know	158	38.5
No	169	41.2
Yes	83	20.2
Do you know that the local injections of Botox could cause side effects like allergic reactions?		
I don't know	171	41.7
No	101	24.6
Yes	138	33.7
Do you know that handling Botox injections by unskilled/non-authorized persons may place patients at risk for potentially devastating consequences?		
I don't know	228	55.6
No	71	17.3
Yes	111	27.1
Do you know that the most feared adverse effect is temporary unwanted muscle weakness/paralysis of nearby musculature caused by the action of the toxin?		
I don't know	161	39.3
No	120	29.3
Yes	129	31.5
Do you know Botulinum toxin should not be used in patients with neuromuscular transmission disorders?		
I don't know	114	27.8
No	194	47.3
Yes	102	24.9
Do you know that Botulinum toxin should not be used along with concomitant use of aminoglycoside antibiotics and spectinomycin because these drugs may potentiate the effect of the toxin?		
I don't know	119	29.0
No	207	50.5
Yes	84	20.5
Do you know that Botulinum toxin should not be used in patients with dysphagia?		
I don't know	115	28.0
No	194	47.3
Yes	101	24.6
Do you know that Botulinum toxin therapy should be avoided during pregnancy?		
I don't know	79	19.3
No	113	27.6
Yes	218	53.2
Do you know that Botulinum toxin therapy should be avoided during lactation?		
I don't know	73	17.8
No	132	32.2
Yes	205	50.0
Do you know that Botulinum toxin therapy could be used in pediatric patients?		
I don't know	109	26.6
No	188	45.9
Yes	113	27.6
Do you know that the effects of botulinum toxin are temporary?		
I don't know	89	21.7
No	129	31.5
Yes	192	46.8
Do you know that Botox treatment is not a cure for any disease?		
I don't know	80	19.5
No	126	30.7
Yes	204	49.8
Do you know that there should be careful monitoring in children as it might alter cell functions such as axonal growth?		
I don't know	101	24.6
No	147	35.9
Yes	162	39.5
Do you know that the weakness induced by injection with botulinum toxin usually lasts about three months?		
I don't know	98	23.9
No	163	39.8
Yes	149	36.3

Among the participants in the age group between 20 and 25, 110 (26.8%) were knowledgeable about the use of botulinum toxin in cosmetic injections. In comparison, only 6 (1.4%) between the age groups 50-55 had knowledge with a recorded statistical significance of (p=0.0001). Moreover, 220 (53.7%) females knew about these procedures in contrast to the male population, which was 42 (10.2%) (p=0.844). Exactly 188 (45.8%) of single individuals were familiar with the use of cosmetic injections as compared to the divorced group (p=0.001) (Table 4).

**Table 4:** Relationship between the socio-demographic factors and knowledge about the use of Botulinum toxin injections in cosmetic procedures Exact probability test; *p<0.05 (significant)

Factors	Yes		No		Maybe		P-value
	Number	%	Number	%	Number	%	
Age in years							
15–20	76	18.5	34	8.2	9	2.1	0.0001*
20–25	110	26.8	27	6.5	12	2.9	
25–30	18	4.3	6	1.4	5	1.2	
30–35	13	3.1	20	4.8	0	0	
35–40	17	4.1	5	1.2	0	0	
40–45	17	4.1	7	1.7	3	0.7	
45–50	5	1.2	17	4.1	0	0	
50–55	6	1.4	0	0	0	0	
Gender							
Female	220	53.7	95	23.1	26	6.3	0.844
Male	42	10.2	21	5.1	6	1.4	
Nationality							
Saudi	247	60.2	106	25.8	32	7.8	0.180
Non-Saudi	15	3.6	10	2.4	0	0	
Education							
Secondary	35	8.5	16	3.9	7	1.7	0.196
Higher secondary	35	8.5	6	1.4	1	0.2	
Bachelors	167	40.7	82	20.0	22	5.3	
Masters	13	3.1	5	1.2	0	0	
Doctorate	12	2.9	7	1.7	2	0.4	
Occupation							
Student	176	42.9	57	13.9	26	6.3	0.0001*
Employee	66	16.1	35	8.5	6	1.4	
Self-employed	14	3.4	0	0	0	0	
Marital status							
Single	188	45.8	67	16.3	21	5.1	0.001*
Married	69	16.8	43	10.4	6	1.4	
Divorced	2	0.4	2	0.48	0	0	
Widow/widower	3	0.7	4	0.9	5	1.2	

Table 4 illustrates the relationship between sociodemographic factors and knowledge of cosmetic procedures. No significant correlation was found between age, gender, marital status, education level, and knowledge of cosmetic procedures.

## Discussion

Globally, both men and women are increasingly undergoing cosmetic operations. According to the International Society of Aesthetic Plastic Surgeons (ISAPS), Saudi Arabia ranks among the top 25 countries for the highest rates of cosmetic treatments and is also within the top 30 countries with the largest number of plastic surgeons. This study represents the first assessment of knowledge, attitudes, and awareness regarding the use of botulinum toxin in cosmetic injections among the general population in Arar, Saudi Arabia. The findings indicated that 63.9% of participants exhibited a solid understanding of botulinum toxin use in aesthetic procedures. This is significantly higher than an earlier global study among Nigerian university students, which found only 4.2% had reasonably good knowledge [22]. This suggests that there is a considerable level of awareness and familiarity with the use of this neurotoxin for aesthetic purposes within the Arar population. The high level of awareness may be attributed to factors such as increased accessibility to information, the growing popularity of cosmetic procedures, and the prevalence of these treatments in the local healthcare landscape.

However, only 15% of participants expressed approval for aesthetic procedures, which is notably lower than in other regions of Saudi Arabia. This is likely due to the more conservative societal norms in this region. Nevertheless, 28% of participants acknowledged that botulinum toxin is one of the most poisonous substances used in cosmetic injections. This finding highlights the need for continued education, strict safety protocols, and regulatory oversight to ensure the safe and responsible use of this potent neurotoxin in cosmetic procedures.

We found that participants who were single, young, and female had the highest perceptions of cosmetic operations out of the three groups we looked at. Societal norms, media influences, and cultural expectations may contribute to women's heightened interest and positive attitudes towards aesthetic procedures, particularly in the context of facial rejuvenation and wrinkle reduction. These kinds of forces could account for the gender gap that is seen. This intersectionality of demographic factors suggests that these individuals may be the most receptive target audience for cosmetic service providers in the Arar region. Likewise, earlier research in Riyadh [23] and worldwide research by Markey and Markey [24] also discovered that Botox treatments are more likely to be accepted by women.

Just 15% of our participants had planned to undergo botulinum toxin treatment in cosmetic procedures. On the other hand, the United States has a 155% cosmetic surgery rate [25], whereas a significant 55.4% of Saudi women have had cosmetic surgery [26]. The reason for this notable discrepancy is the preference for invasive procedures like breast augmentation and liposuction among American women. In this study, many participants agreed to the use of botulinum toxin for dental procedures and pain.

A study conducted in Bushehr, Southern Iran, in 2012 on factors influencing patients' decisions to undergo cosmetic surgery revealed that demographic characteristics significantly impact these decisions. Specifically, women and individuals aged 30 to 45 showed a greater inclination toward cosmetic procedures [27]. The increased likelihood of those in the 30 to 45 age group opting for cosmetic surgery may stem from various factors, including the emergence of visible signs of ageing, shifts in self-perception, and a desire to maintain a youthful appearance. The study found a significant association between participants' attitudes and age (p = 0.001).

The Acceptance of Cosmetic Surgery Scale (ACSS) revealed a significant difference in scores between genders, with female patients scoring higher than male patients (p = 0.001). Additionally, there was a significant correlation between age and the decision to undergo cosmetic procedures (p = 0.0001), with the 20-25 age group demonstrating the most knowledge about the use of botulinum toxin in cosmetic treatments. The increased knowledge and potential acceptance of botulinum toxin-based cosmetic procedures among the younger generation may have broader societal implications, such as shifting perceptions of beauty, self-care, and the normalization of cosmetic interventions. However, targeted educational campaigns and awareness programs may be necessary to address any knowledge gaps among older individuals and ensure a well-informed populace [28].

A retrospective analysis of 4,550 cosmetic surgeries conducted in China in 2016 found that the most popular age group was patients aged 19 to 34, who made up 76.9% of the total [29].

Another notable finding in our study is that not the majority of respondents had awareness about the harmful effects of botulinum toxin. About 20% of participants knew that local injections of botulinum toxin could cause ptosis (droopy eyelids), a heavy brow, or a frozen face in terms of facial aesthetics. Informing patients about the nature of the substance being used, its potential risks, and the importance of seeking treatment from qualified professionals can help empower patients to make informed decisions and mitigate the risks associated with botulinum toxin injections [30]. A large number of participants lacked knowledge about contraindications and the adverse effects of injections, which highlights the need for increased education. It highlights the need for strict safety protocols, regulation, and oversight in the administration of botulinum toxin injections. This awareness underscores the importance of ensuring that only qualified and licensed professionals perform these procedures and that appropriate safety measures are in place to minimize the risks of complications or adverse effects.

Strengths and limitations

This study is the first to be carried out in the city of Arar, Saudi Arabia, with the intention of evaluating the general public's knowledge, attitudes, and awareness of botulinum toxin use in cosmetic injections. The study design leveraged a multifaceted approach to social networks, engaging participants from a variety of interpersonal and community affiliations.

The primary limitation of this study was its cross-sectional survey design and reliance on self-reported data, which may not accurately capture the dynamics of cosmetic trends. Furthermore, the research's sample was limited to people who were present in the city, Arar, which reduced the generalizability of the results to all Saudi Arabian citizens. We advise carrying out additional investigations in other regions of Saudi Arabia, as variations in customs and culture could result in different outcomes.

## Conclusions

This study reveals a noteworthy level of awareness regarding botulinum toxins among the population, with 63.9% demonstrating knowledge about their use in cosmetic procedures. However, despite the stark contrast between this awareness and the limited understanding of the potential adverse effects, only 20.2% of participants acknowledged these risks, highlighting a critical gap in public knowledge. The predominance of young individuals, particularly females and unmarried people, suggests a demographic that is increasingly engaging with cosmetic interventions. This trend emphasizes the importance of targeted educational initiatives aimed at these groups, who may be more susceptible to the allure of cosmetic enhancements without fully comprehending the associated risks. We conclude that to foster a safer environment for those considering cosmetic procedures, it is essential to implement comprehensive education and awareness campaigns. These initiatives should not only focus on the benefits of botulinum toxins but also thoroughly address potential side effects, ensuring that individuals are equipped with the necessary information to make informed decisions.

## Appendices

**Table 5:** Demographic details of participants Busaadia et al. [21]

S.no	Variables		Comments
1	Age in years	18–20	
		20–35	
		35–40	
		40–45	
		50–55	
		60>	
2	Gender	Male	
		Female	
3	Weight in kg	Less than 40	
		40–50	
		51–60	
		61–70	
		71–80	
		More than 80	
4	Height in cm	Less than 140	
		141–150	
		151–160	
		161–170	
		More than 170	
5	Nationality	Saudi	
		Non-Saudi	
6	Are you a Saudi national, and do you belong to Arar City of the Northern Province region?	Yes	
		No	
		Foreigner	
7	Level of education	Primary	
		Secondary	
		Higher secondary	
		Bachelor	
		Master	
		Others	
8	What is your occupation?	Student	
		Employee	
		Self-employed	
		Private business	
		Any other mention	
9	Marital status	Single	
		Married	
		Widow/Widower	
		Divorced	
10	Do you plan to have or have undergone Botox treatment?	Yes	
		No	
		Not sure	

**Table 6:** Participant’s knowledge and attitude towards botulinum toxin use Busaadia et al. [21]

S.No	Questions related to participants knowledge and attitude towards Botox toxins usage	Yes	No	Neutral
1	Do you know that botulinum toxin is one of the most poisonous biological substances known?			
2	Do you know about Botox treatments used in cosmetic clinics?			
3	Have you or any of your family members taken Botox treatment for any type of disease?			
4	If yes, please mention the name: 1. Dystonias 2. Spasticity 3. Autonomic Disorders 4. Urologic Pathologic Conditions 5. Pain 6. Gastroenterology disorders 7. Dental treatment			
5	Have you or any of your family member has used Botox treatment to reduce all wrinkles in all skin areas of the face and the neck?			
6	Do you know that the best results are obtained when BoNT is combined with filler substances to correct non dynamic aspects of the skin lines?			
7	Are you aware how to safely dispose of leftover needles after using them?			
8	Are you aware botulinum toxins vials need to be stored according to the protocol approved by their manufacturer?			
9	Do you think that buying needles without a prescription is against law?			
10	Do you think that for those who wish to take Botox injections should contact a dermatologist instead of non-specialized workers in cosmetic centers?			
11	Are you aware of the high cost of Botox treatment?			
12	Are you aware that after two to three weeks of having Botox treatment the medicine starts showing desired results?			
13	Are you aware that patients having frequent booster injections seem to have a higher risk of developing antibodies and tolerance?			
14	Are you aware that bruising can occur, particularly if a small vein is lacerated or a patient is taking aspirin, vitamin E, or NSAIDs?			
15	Do you know that these Botox injections can be repeated to maintain the desired results?			

**Table 7:** Creating awareness regarding Botox toxins adverse effects and misuse Busaadia et al. [21]

S.No		Yes	No	I don’t know
1	Do you know that the possibility of local diffusion or potential leakage of Botulinum toxin into the systemic circulation exists?			
2	Do you know that the local injections of Botulinum toxin could cause ptosis (droopy eyelids) and heavy brows, or a frozen face in facial aesthetics?			
3	Do you know that the local injections of Botox could cause side effects like allergic reactions?			
4	Do you know that handling of BoNTs by unskilled/non-authorized persons or the use of counterfeit or unapproved agents that have permeated the market worldwide, or their combination, may place patients at risk for potentially devastating consequences?			
5	Do you know that the most feared adverse effect is temporary unwanted muscle weakness/paralysis of nearby musculature caused by the action of the toxin?			
6	Do you know that botulinum toxin should not be used in patients with neuromuscular transmission disorders?			
7	Do you know that botulinum toxin should not be used along with concomitant use of aminoglycoside antibiotics and spectinomycin because these drugs may potentiate the effect of the toxin?			
8	Do you know that botulinum toxin should not be used in patients with dysphagia?			
9	Do you know that botulinum toxin therapy should be avoided during pregnancy?			
10	Do you know that botulinum toxin therapy should be avoided during lactation?			
11	Do you know that botulinum toxin therapy could be used in pediatric patients?			
12	Do you know that the effects of botulinum toxin are temporary?			
13	Do you know that Botox treatment is not a cure for any disease?			
14	Do you know that there should be careful monitoring in children as it might alter cell functions such as axonal growth?			
15	Do you know that the weakness induced by injection with botulinum toxin usually lasts about three months?			
